# Conventional and molecular cytogenetics of human non-medullary thyroid carcinoma: characterization of eight cell line models and review of the literature on clinical samples

**DOI:** 10.1186/1471-2407-8-371

**Published:** 2008-12-16

**Authors:** Franclim Ricardo Ribeiro, Ana Margarida Meireles, Ana Sofia Rocha, Manuel Rodrigues Teixeira

**Affiliations:** 1 Department of Genetics, Portuguese Oncology Institute, Porto, Portugal; 2 Institute of Molecular Pathology and Immunology of the University of Porto, Porto, Portugal; 3 Department of Biochemistry, Medical Faculty, University of Porto, Porto, Portugal; 4 Institute of Biomedical Sciences Abel Salazar (ICBAS), University of Porto, Portugal

## Abstract

**Background:**

Cell lines are often poorly characterized from a genetic point of view, reducing their usefulness as tumor models. Our purpose was to assess the genetic background of eight commonly used human thyroid carcinoma models and to compare the findings with those reported for primary tumors of the gland.

**Methods:**

We used chromosome banding analysis and comparative genomic hybridization to profile eight non-medullary thyroid carcinoma cell lines of papillary (TPC-1, FB2, K1 and B-CPAP), follicular (XTC-1) or anaplastic origin (8505C, C643 and HTH74). To assess the representativeness of the findings, we additionally performed a thorough review of cytogenetic (n = 125) and DNA copy number information (n = 270) available in the literature on clinical samples of thyroid carcinoma.

**Results:**

The detailed characterization of chromosomal markers specific for each cell line revealed two cases of mistaken identities: FB2 was shown to derive from TPC-1 cells, whereas K1 cells have their origin in cell line GLAG-66. All cellular models displayed genomic aberrations of varying complexity, and recurrent gains at 5p, 5q, 8q, and 20q (6/7 cell lines) and losses at 8p, 13q, 18q, and Xp (4/7 cell lines) were seen. Importantly, the genomic profiles were compatible with those of the respective primary tumors, as seen in the meta-analysis of the existing literature data.

**Conclusion:**

We provide the genomic background of seven independent thyroid carcinoma models representative of the clinical tumors of the corresponding histotypes, and highlight regions of recurrent aberrations that may guide future studies aimed at identifying target genes. Our findings further support the importance of routinely performing cytogenetic studies on cell lines, to detect cross-contamination mishaps such as those identified here.

## Background

Thyroid cancer represents the most frequent endocrine malignancy in humans, ranging from the more differentiated carcinomas with papillary (80–90% of all occurrences) or follicular (5–10%) histotypes, to the rare and clinically more aggressive anaplastic carcinomas (2–5%)[1]. Many of the genetic events leading to the oncogenic and metastatic potential of these distinct malignancies have already been identified [2]. Papillary thyroid carcinomas (PTC) frequently harbor activating rearrangements of *RET *(8–33%) or *NTRK1 *(5–15%) genes, both coding for receptor tyrosine kinases that exert control over a wide range of transcription factors [3]. Some PTC may display, instead, mutually exclusive point mutations in *RAS *(0–21%) or *BRAF *(29–83%), leading to the constitutive activation of the RAS-RAF-MEK-ERK signaling pathway [4]. Follicular thyroid carcinomas (FTC), on the other hand, are known to harbor *PAX8-PPARγ *gene fusions (25–60% of the cases) and also mutations in *RAS *(21–50%) [4]. Undifferentiated thyroid carcinomas (UTC) may display *RET *rearrangements (less than 10%) or point mutations in *RAS *(20–60%) or *BRAF *(0–63%), as well as mutations in *TP53 *(up to 60%), which is in accordance with the hypothesis that most UTC originate from well-differentiated lesions through the multi-step accumulation of genetic aberrations [2,4]. Deregulation of the phosphatidylinositol 3-kinase (PI3K)/Akt pathway through amplification or activating mutations at the catalytic subunit of PI3K (*PIK3CA*, 12–23%), or inactivating mutations of key inhibitors such as *PTEN *(up to 16%), also plays a relevant role in UTC etiology [5].

The molecular mechanisms behind a considerable proportion of thyroid carcinomas remain nevertheless unclear, and extensive effort has been placed in creating *in vitro *and *in vivo *models of thyroid carcinogenesis. Several cell lines derived from PTC, FTC, and UTC carcinomas have been established and are widely used to evaluate novel oncogenic events or molecular markers with diagnostic, prognostic and/or therapeutic potential. The genetic content of many of these cell lines, however, is poorly or only partially characterized, making it difficult to assess the pathogenetic role of specific gene rearrangements in the absence of a general picture of the genomic background of each cell line.

To contribute to the genetic characterization of the in vitro models of non-medullary thyroid carcinogenesis, we performed chromosome banding analysis and chromosomal comparative genomic hybridization (CGH) on eight human thyroid carcinoma cell lines originating from papillary carcinomas (TPC-1, FB2, K1, and B-CPAP), follicular carcinoma (XTC-1) or undifferentiated carcinomas (C643, 8505C, and HTH74). We additionally review the karyotypic and CGH information available in the literature for these three thyroid carcinoma histotypes, in order to assess the primary tumor representativeness of these cell lines.

## Methods

### Cell lines and culture conditions

A total of eight human thyroid carcinoma cell lines, originally thought to derive from distinct patients, were assessed in the present study (Table 1). All cell lines were maintained in RPMI medium with Glutamax (GIBCO BRL – Invitrogen Corporation, Carlsbad, CA) supplemented with 10% fetal bovine serum (FBS) and antibiotics, except for K1 which was cultured in Dulbecco's modified Eagle's medium (GIBCO BRL – Invitrogen Corporation, Carlsbad, CL) supplemented with 10% FBS and antibiotics. Cells were cultured as monolayer in a humidified atmosphere (5% carbon dioxide) at 37°C.

**Table 1:** Tumor origin and source of the eight thyroid cell lines analyzed in this study

**Cell line**	**Tumor origin**	**Gender**	**Source**	References*
TPC-1	Papillary carcinoma	Female	Prof Dumont JE; Prof Mareel M; Laboratory of Experimental Cancerology, Ghent University Hospital, Ghent, Belgium	**Tanaka *et al*. 1987 **[10] Jossart *et al*. 1995 [15] Miura *et al*. 2003 [20] van Staveren *et al*. 2007 [21]
FB2 (*TPC-1*)	Papillary carcinoma	Female	Prof. Santoro M; Centro di Endocrinologia ed Oncologia Sperimentale, Napoly, Italy	**Basolo *et al*. 2002 **[16]
K1 (*GLAG-66*)	Papillary carcinoma	Male	Prof. Wynford-Thomas D; Department of Pathology, University of Wales College of Medicine, Cardiff, U.K.	**Antonini *et al*. 1993 **[19] Challeton *et al*. 1997 [18]
B-CPAP	Papillary carcinoma	Female	Prof Mareel M; Laboratory of Experimental Cancerology, Ghent University Hospital, Ghent, Belgium	**Paulin *et al*. 1992 **[29] Corso *et al*. 2002 [22] Dettori *et al*. 2004 [23] van Staveren *et al*. 2007 [21]
XTC-1	Follicular carcinoma (Hürthle cell)	Female	Prof. Savagner F; Laboratoire de Biochimie et Biologie Moleculaire, Faculte de Medecine, Angers, France	**Zielke *et al*. 1998 **[30]
HTH74	Undifferentiated carcinoma	Female	Prof Mareel M; Laboratory of Experimental Cancerology, Ghent University Hospital, Ghent, Belgium	**Heldin *et al*. 1991 **[31] Lee *et al*. 2007 [24]
C643	Undifferentiated carcinoma	Male	Prof Mareel M; Laboratory of Experimental Cancerology, Ghent University Hospital, Ghent, Belgium	**Mark *et al*. 1987 **[32] Lee *et al*. 2007 [24]
8505C	Undifferentiated carcinoma	Female	Prof Mareel M; Laboratory of Experimental Cancerology, Ghent University Hospital, Ghent, Belgium	**Ito *et al*. 1992 **[33] van Staveren *et al*. 2007 [21] Lee *et al*. 2007 [24]

* Only papers detailing the establishment (references in bold) or providing relevant genetic information for each cell line are indicated.

### Chromosome banding analysis

Upon attaining optimal cellular density, cultures were harvested and cells divided into two tubes. One tube was processed for cytogenetic analysis, and metaphases were GTG-banded according to standard procedures. The second tube was used for DNA extraction. Clonality criteria and karyotype description followed the International System for Human Cytogenetic Nomenclature (ISCN) 2005 [6].

### Comparative genomic hybridization

Chromosomal CGH was performed as previously described [7]. Briefly, test (cell lines) and reference (peripheral blood lymphocytes from healthy donors) DNA was extracted using standard methods and labeled in nick translation reactions using SpectrumGreen- and SpectrumRed-conjugated nucleotides (Vysis, Downers Grove, IL). The same amount of differentially labeled cell line and reference DNA (1 μg) was then mixed with Cot-1 DNA (30 μg) and hybridized onto commercially available, normal metaphase slides (Vysis, Downers Grove, IL). Hybridization took place for 2–3 days at 37°C in a moist chamber, after which excess probe was washed off and DAPI counterstain was applied. Analysis was performed using a Zeiss Axioplan fluorescence microscope (Zeiss, Oberkochen, Germany) and a CytoVision system version 3.0 (Applied Imaging, Santa Clara, CA). Scoring was based on dynamic standard reference intervals generated based on data from 10 normal versus normal hybridizations. Aberrations were scored whenever the case profile and the standard reference profile at 99% confidence did not overlap. Amplifications were scored whenever the 99% confidence interval for a given sample crossed the 1.75 threshold. Description of CGH copy number changes followed the recommendations of the ISCN 2005 [6].

### Literature review

Karyotypic information for non-medullary thyroid carcinoma samples was obtained from Mitelman database of chromosomal aberrations in cancer [8]. Cases were subdivided according to the three major histotypes (papillary, follicular and anaplastic) and the modal number, the total number of chromosome aberrations, and all breakpoints in each sample were annotated. Chromosomal CGH information was obtained from thyroid carcinoma publications listed in the Progenetix database [9], and copy number diagrams were generated for each of the three histotypes.

### Fluorescence in situ hybridization

Locus-specific probes targeting chromosomal regions 5p15 and 5q33-34 were applied to C643 and HTH74 metaphase spreads in order to clarify the origin of several chromosomal markers. Sample processing, hybridization, and analysis were performed according to standard protocols. Briefly, the same nuclear suspensions prepared for cytogenetic studies were dropped onto glass slides, air dried and pre-treated in 2 × SSC/0.5% Igepal (Sigma-Aldrich, Steinheim, Germany) at 37°C for 30 minutes, after which slides were dehydrated in a series of ethanol. A SpectrumOrange labeled CSF1R probe (5q33-34) and a SpectrumGreen labeled D5S23/D5S721 probe (5p15.2) were applied to both cell line samples (Vysis, Downers Grove, IL). Slides were placed in a Hybrite denaturation/hybridization system (Vysis, Downers Grove, IL) and co-denatured at 75°C for 3 minutes. Hybridization took place for 18 h at 37°C, followed by post-hybridization washes in 0.4 × SSC/0.3% Igepal at 72°C for 2 minutes and 2 × SSC/0.1% Igepal at room temperature for 1 minute. Slides were counterstained with DAPI (Vector Laboratories, Burlingame, CA).

## Results

**Figure 1:**
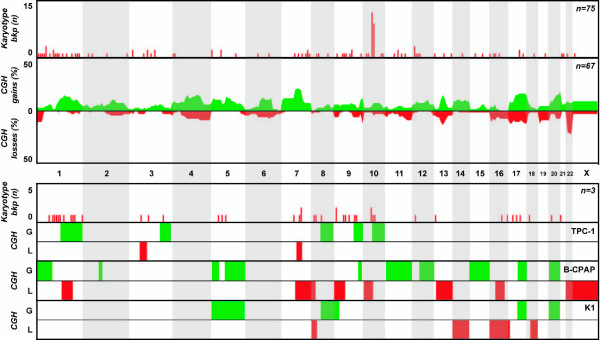
**Overview of genomic findings in papillary thyroid carcinoma**. Karyotype and CGH copy number information from clinical samples reported in the literature (top two charts) and from the three papillary cell lines included in this study (bottom charts) are included.

**Figure 2:**
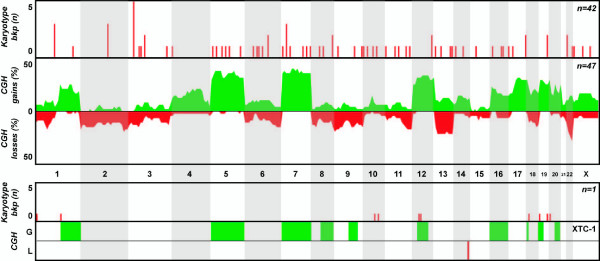
**Overview of genomic findings in follicular thyroid carcinoma**. Karyotype and CGH copy number information from clinical samples reported in the literature (top two charts) and from the follicular cell line included in this study (bottom charts) are included.

**Figure 3:**
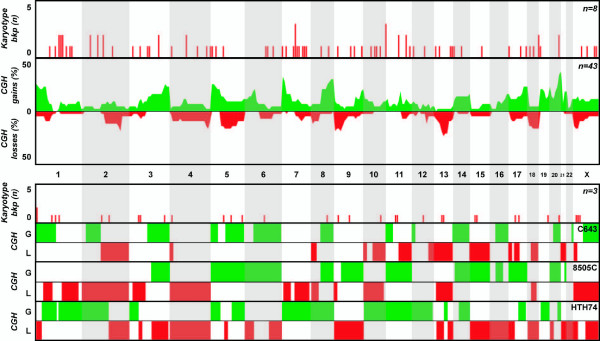
**Overview of genomic findings in anaplastic thyroid carcinoma**. Karyotype and CGH copy number information from clinical samples reported in the literature (top charts) and from the three anaplastic cell lines included in this study (bottom charts) are included.

A detailed description of the cytogenetic and molecular cytogenetic findings obtained in the eight cell lines is presented in Table 2, and representative karyograms are supplied as supplementary files (Additional files 1, 2, 3, 4, 5, 6, 7, 8). Interestingly, the cytogenetic features shared by cell lines TPC-1 and FB2 show that the latter is derived from the former through cross-contamination (Additional files 9, 10), thus reducing to seven the number of independent cell lines studied. To facilitate visual comparisons between findings on different cell lines and to integrate these data with the available genetic information on primary tumor samples, diagrams integrating G-banding and CGH information were generated for the three assessed thyroid tumor histotypes (Figures 1, 2, 3).

**Table 2:** Cytogenetic and chromosomal CGH findings in seven unique thyroid cell lines

**Cell line**	**Karyotype**	**CGH**
TPC-1	49,XX,der(1)t(1;10;21)(1pter→1q31::21q21→21qter), +der(1)t(1;3)(p13;p21),der(3)t(3;9)(p11;q22), del(7)(q22q31),+i(8)(q10),der(10)t(1;10;21) (10q11.2→10pter::1q31→1qter),+der(10)inv(10) (q11.2q21)t(3;10)(q25;p11),der(21)t(1;10;21) (21pter→21q21::10q21.2→10q11.2::10q21.2→10qter)[25]	rev ish enh(1)(q),dim(3)(p12p21),enh(3)(q24q29), dim(7)(q21q31),amp(8)(q),enh(9)(q22q34),enh(10)(q)
K1 *(GLAG-66)*	87~93,XXYY,der(1)t(1;9;9)(9pter→9p11::1p11→1q44::9p12→9pter)x2,i(5)(p10),i(5)(q10),der(7)t(1;7) (p31;q31),der(8)t(8;9)(p22;p23)x2,+der(8)t(1;8) (p11;q11),del(9)(p11)x2,del(9)(p12)x2,-13,-14,-16, -17,der(18)t(17;18)(q21;q12)x2,+20 [cp25]	rev ish enh(5),dim(8)(p21p23),enh(8)(q), amp(9)(p22p24),dim(14)(q),dim(16),dim(17)(p13), enh(17)(q21q25),dim(18)(q12q23),enh(20)
B-CPAP	70~74,XX,-X,der(1)t(1;1)(p12;p21),der(5)t(5;9) (q11;q12), +dic(5;13)(p12;q12),+i(5)(p10),-7, der(8)t(5;8)(q13;p21),der(9)t(9;9)(p23;q31), der(10)t(1;10)(q25;p11),der(10)t(1;10)(q11.2;p11.2) del(1)(q21q32),+11,+dic(12;20)(p11;p11),-13,+15, add(16)(q22),der(17)inv(17)(p13q11)inv(17)(q11q23),+20,+20,der(20)t(7;20)(p11;p11),-22 [cp25]	rev ish dim(X),enh(1)(p21p36),dim(1)(q21q31), enh(2)(p11p12),enh(5)(p12p15),enh(5)(q13q35), dim(7)(q),dim(8)(p22p23),dim(9)(p),enh(9)(q32q33), dim(10)(p),enh(11),enh(12)(q12q24),dim(13)(q), enh(15)(q),dim(16)(q11q22),enh(17)(q21q25),amp(20),dim(22)(q)
XTC-1	51~58,XX,add(1)(p36),+5,+7,+7,+der(8;12)(q10;q10)x2,dup(10)(q22q24),add(12)(q14),del(12)(q13),+16, +del(18)(q11),+19,+19,der(19)t(1;19)(q21;p13) del(19)(q13)x2,+20,add(20)(p11)x2 [cp25]	rev ish enh(1)(q),enh(5),amp(7),amp(8)(q), enh(9)(q12q31),enh(9)(q33q34),enh(12)(q11q21), amp(12)(q11q15),dim(14)(q32),enh(16),enh(18)(p), enh(19)(p13q13),enh(20)(q),amp(20)(q13)
HTH74	57~60,X,der(X)t(X;17)(p11;q12),+add(1)(p13), der(1)t(X;1)(p21;p36),der(2)t(2;20)(q21;q13),t(3;8) (p21;q22)x2,dup(5)(q22q35)x2,+i(5)(p10)x2,+7,+7, +8,+8,+10,+del(11)(q13)x2,+12,dup(13)(q22q31)x2, der(14)t(14;19)(p11;q13),add(15)(q15)x2, der(17)t(11;17)(q12;q22),+19,der(19)t(15;19) (q21;q13)x2,+20 [cp25]	rev ish dim(X)(p),dim(X)(q22q28),dim(1)(p34p36), enh(1)(p33q44),enh(2)(p25q14),dim(2)(q21q37), amp(3)(p22p26),dim(3)(p14p21),dim(3)(q13q28), dim(4),amp(5)(p),dim(5)(q13q14),enh(5)(q22q35), amp(5)(q23q35),dim(6)(p21p25),dim(6)(q21q27), amp(7),amp(8),dim(9),enh(10),enh(11),amp(11)(p), enh(12)(p13q23),dim(13)(q12q21),enh(13)(q22q31), dim(13)(q32q34),enh(14)(q21q32),dim(15)(q),dim(16), dim(17)(p),enh(17)(q21q24),amp(17)(q21q22),dim(18),enh(19)(p12q13),amp(19)(q13),dim(20)(p12p13), enh(20)(q12q13),dim(21)(q),dim(22)(q)
C643	72~82,YY,add(X)(p?),der(X)add(X)(q?)add(X)(p?), der(1)t(1;5)(p36;q13),der(1;4)(p10;q10),+del(1)(q10), -2,add(2)(q11),+der(9;11)(q10;q10),add(4)(p15), +i(5)(p10),der(8;15)(q10;q10)x2,+der(8;13) (q10;q10),-9,-10,add(10)(q25),-11,dic(11;11)dup(11) (pter→q13::q23→q14::q14→q23::p15),der(?)t(?;6) (?;q15),mar1,mar2,mar3 [cp25]	rev ish enh(X)(p22),dim(X)(p11p21),enh(X)(q21q28), enh(1)(p),amp(1)(p11p31),enh(2)(p11p22),dim(2)(q), enh(3)(q),dim(4)(p15p16),enh(5)(p),enh(5)(q14q35), amp(5)(q21q33),enh(6)(p12q27),dim(8)(p21p23), enh(8)(q),amp(8)(q11q23),dim(10)(p14p15), dim(10)(q),enh(11)(p),dim(11)(q13q25),enh(12) (p12p13),dim(12)(q22q24),dim(13)(q),enh(14)(q), dim(15)(q),enh(16)(q),dim(17)(p12p13),dim(17) (q11q21),dim(18)(q12q23),enh(20)(p11q13),dim(21)(q)
8505C	59~63,X,-X,der(X)t(X;9)(p22;q13),-1,del(1)(q12), del(1)(p11),-2,der(3)del(p14)del(q27),-4,+i(5)(p10), -7,i(8)(q10),del(9)(p21),-10, del(12)(q15),-13,-14,-18, -19,+20,+20,-21,-22,+mar [cp25]	rev ish dim(X),dim(1)(p13p32),dim(1)(q22q43),dim(2), dim(3)(p12p24),enh(3)(q),dim(4),amp(5)(p),enh(5)(q), enh(6),dim(7)(p13p21),dim(7)(q11q35),dim(8) (p12p23),amp(8)(q),dim(9)(p23p24),enh(9)(p13q34), dim(10)(p15q25),enh(11),dim(13)(q),enh(14)(q12q32), enh(15)(q),enh(16)(q11q21),enh(17),dim(18)(q12q22), amp(20),enh(21)(q22),dim(22)(q12q13)

Karyotype and CGH description follow the guidelines of the ISCN (2005) [6].

### Cell line overview

All cell lines displayed numerical and structural aberrations of varying complexity, some of which could not be completely identified by classical cytogenetic analysis. Isochromosomes 5p (3 cell lines) and 8q (2 cell lines) were seen recurrently, whereas similar breakpoints were seen by chromosome banding at 1p36 (3 events), 5q13 (3 events), and 3p21, 7q31, 8p22, 9p23, and 10p11 (2 events each). Interestingly, distinct cytogenetic aberrations resulted in identical net gain and/or loss of genomic material at several chromosomal regions, as seen by CGH. In particular, gains at 5p, 5q, 8q, and 20q were seen in 6/7 cell lines, whereas gains at 11p and 17q and losses at 8p, 13q, 18q, and Xp were seen in 4/7 cell lines (Figures 1, 2, 3). Recurrent amplification events were seen at 8q (4 cell lines), 20q (3 cell lines), and 5p, 5q, 7pq, and 20p (2 cell lines each, Table 3). Gains at 14q21q32 and losses at 2q21q37 and 4p15p16 were seen exclusively in the three UTC cell lines (HTH74, C643 and 8505C), whereas none of the imbalances detected in the three papillary-derived cell lines (TPC-1, K1 and B-CPAP) was exclusive of that subset. XTC-1 (follicular Hürthle-cell tumor) shared copy number imbalances with papillary and/or UTC cell lines, and so no histotype-related copy number features can be highlighted.

**Table 3:** Overview of recurrent CGH findings in seven thyroid carcinoma cell lines

	Frequency	SRO
Gains		
5p	6/7	5p12p15
5q	6/7	5q22q35
8q	6/7	8q11q24
20q	6/7	20q12q13
11p	4/7	11p12p15
17q	4/7	17q21q24
Losses		
8p	4/7	8p22p23
13q	4/7	13q12q21 13q32q34
18q	4/7	18q12q22
Xp	4/7	Xp11p21
Amp		
8q	4/7	8q11q23
20q	3/7	20q11q13
5p	2/7	5p12p15
5q	2/7	5q23q33
7pq	2/7	7p22q36
20p	2/7	20p11p13

For gains and losses, only regions seen in more than 3 cell lines are provided. Abbreviations: SRO, smallest region of overlap; Amp, amplifications.

### Karyotype review

A total of 125 abnormal karyotypes from independent samples of patients with non-medullary thyroid carcinoma were retrieved from Mitelman's database (25 literature references). Tumors with papillary differentiation represented the largest group (n = 75) and encompassed mostly samples with classical morphology, although at least 8 tumors were classified as follicular variants of papillary carcinoma. In general, papillary tumors displayed very simple karyotypes with a modal number of 46 chromosomes per sample, and an average of 2.4 aberrations per tumor. Whereas few recurrent structural aberrations were seen, 12 cases displayed rearrangements at 10q11 (*RET *locus), and indeed seven samples displayed the typical inv(10)(q11.2q21) generating the *RET*/PTC1 chimera, two samples displayed the t(7;10)(q35;q21) variant (*RET*/PTC6), and the t(1;10)(p13;q11) and t(10;17)(q11;q23) were seen in one sample each (*RET*/PTC7 and *RET*/PTC2, respectively). Loss of Y (n = 8), loss of 22 (n = 8) and gain of chromosome 5 (n = 5) and/or 7 (n = 10) were numerical aberrations recurrently observed. One of the eight papillary tumors with follicular growth pattern displayed the t(2;3)(q13;p25) associated with follicular tumors.

Within the group of tumors with follicular histotype (n = 42), the modal number of chromosomes per sample was also 46, but an average of 5.9 aberrations per tumor was observed. Again few recurrent structural aberrations were described, and only two samples displayed the t(2;3)(q13;p25) responsible for the *PAX8-PPARγ *chimera. Additional recurrent breakpoints were seen at 1p13 (n = 3) and 7p15 (n = 3). Numerical aberrations were much more frequent in this subgroup, however, including recurrent gains of chromosome 5 (n = 4), 7 (n = 10), 16 (n = 4) and 20 (n = 4) and losses of 2 (n = 6), 3 (n = 5), 6 (n = 4), 8 (n = 7), 11 (n = 6), 13 (n = 6), 15 (n = 6), 17 (n = 8), 18 (n = 9), 21 (n = 6) and 22 (n = 4).

Karyotypic information for anaplastic thyroid carcinomas is more difficult to interpret, as it is limited to 8 samples displaying very complex and incompletely described karyotypes. The median number of chromosomes per sample was 76, and on average each tumor displayed 16 cytogenetic aberrations. Recurrent translocation breakpoints were seen at 7q11 (n = 3) and 11p15 (n = 3), but no other structural or numerical aberrations were seen in more than one sample.

### CGH review

Fifteen publications dealing with non-medullary thyroid carcinoma samples could be mined for DNA copy number information, providing a total of 270 independent tumors. Of these, 157 (58%) displayed copy number changes. Papillary tumors comprised the main group of samples (n = 158), of which 67 (42%) displayed copy number aberrations. This group generally displayed few aberrations per sample, mostly involving small gains and/or deletions. The distribution of changes across the genome, however, seems to follow a random pattern, as the same chromosomes were often lost or gained in similar proportions in independent studies (Fig. 1). Concerning follicular tumors, 47 out of 58 samples (81%) displayed copy number changes that were clearly non-random. Whole chromosome alterations were particularly frequent, of which gains of 5, 7 and 12 were the most common and losses of chromosomal regions at 2q, 6q, 9p, 11q, 13q, and 22q are also noteworthy for its frequency (Fig. 2). Finally, in the subset of anaplastic tumors, 43 out of 54 samples (80%) displayed copy number aberrations. These complex tumors showed several copy number alterations per sample, affecting almost all chromosomes. However, a clearly non-random pattern of alterations can be seen, in which smaller regions of gains and losses (rather than whole chromosome aberrations) are frequent (Fig. 3). In particular, terminal gains at 1p, 3q, 5p, 7p, 8q and 20q, as well as losses at 5q, 9p, 13q, 18q and Xp, were seen in up to 20% of the samples.

### Fluorescence in situ hybridization

FISH data corroborated the findings obtained by G-banding and CGH regarding chromosome 5 markers in cell lines C643 and HTH74. Whereas considerable variability was seen in nuclei and metaphases from C643 cells, the most representative cellular population displayed signals corresponding to three normal chromosomes five, an i(5)(p10) and a large isochromosome containing several 5q33-34 segments. In HTH74 cells, and in addition to an i(5)(p10), the two large chromosome 5 derivatives showed one extra signal for the CSF1R probe, confirming that a duplication encompassing this region had occurred.

## Discussion

Cell lines are widely used by the scientific community as cellular models in cancer research, providing unique opportunities to perform *in vivo *studies otherwise impracticable in clinical samples. In many cases, however, cell lines are poorly characterized from a genetic point of view, reducing their usefulness as tumor models and making more difficult the recognition of mistaken identities. In our effort to assess the cytogenetic features of eight commonly used human thyroid carcinoma cell lines we came across two such examples. The first unexpected finding occurred when the karyotypes of TPC-1 and FB2 were compared, as several unique cytogenetic abnormalities were shared by the two cell lines. The second example involves cell line K1, which shares the complex cytogenetic markers of cell line GLAG-66. Our data and the review of the literature (discussed below) allow us to conclude that FB2 derived from TPC-1 through a cell culture cross-contamination, whereas K1 derived from GLAG-66 likely due to a similar mishap.

Cytogenetic data on TPC-1 is unexpectedly scarce if one bears in mind that this cell line has been studied for more than 20 years [10]. It is known to harbor an inv(10)(q11.2q21) that fuses the *RET *proto-oncogene at 10q11.2 with the promoter region of the *H4 *gene (10q21), creating the *RET*/PTC1 chimera [11]. Oncogenic fusion events involving *RET *occur in a large percentage of papillary thyroid carcinomas and multiple fusion partners have been identified [12-14]. A more distinguishing feature of this diploid cell line, however, is the complex translocation involving chromosomes 1, 10 and 21 [15]. From a cytogenetic point of view, this t(1;10;21) represents a unique fingerprint for TPC-1 cells, unequivocally confirming the authenticity of our sample. The near-tetraploid cell line FB2 was more recently reported by Basolo *et al*. (2002), who provide a quinacrine mustard staining (QFQ banding) karyogram fully compatible with our findings [16]. Strikingly, two copies of each of the three abnormal chromosomes resulting from the t(1;10;21) are readily identifiable in their Figure 4, together with other structural changes seen in TPC-1, such as a del(7)(q22q31) and an i(8)(q10). It is likely that Basolo *et al*. did not notice the cytogenetic similarities between these cell lines due to the different ploidy and because they misinterpreted the der(10)t(1;10;21) as an inv(10)(q11.2p15.3), which would have a similar morphology under QFQ staining. The authors also state that both TPC-1 and FB2 cell lines were utilized simultaneously in their laboratory and their experimental data on the two cell lines are identical [16]. Since the karyotypes we determined for TPC-1 and FB2 are compatible with the original reports for both cell lines, we must conclude that a cross-contamination mishap occurred during the attempt to establish FB2, which in fact represents a tetraploid population of TPC-1 cells.

Another example of misidentity was found when analyzing K1 cells, which display two copies of a very distinctive chromosome 1 derivative containing several 9p segments (Additional file 3). Whereas Wyllie *et al*. were the first to report the use of K1 cells to characterize several of its molecular features, no cytogenetic information was provided [17]. Two years later, the group that supplied Wyllie and coworkers with the K1 cell line reported its establishment, although again no karyotypic information was given [18]. Interestingly, this group had reported in 1993 the establishment and cytogenetic characterization of a novel near-diploid thyroid cell line named GLAG-66, and the presence of the same unique chromosome 1 derivative in this cell line is very clear from the karyotypic description and figures provided [19]. From a cytogenetic point of view, the complexity of this shared rearrangement clearly confirms that K1 is a tetraploid subpopulation of the GLAG-66 cell line, which was obtained from a metastasis of a well-differentiated papillary thyroid carcinoma.

Our combined chromosome banding and CGH analysis of the remaining cell lines allowed a detailed genomic characterization of their chromosomal changes, and a very high concordance between the two genome screening methodologies was achieved. Our data are also compatible with the existing literature findings available for some of these cell lines, which are scattered across several publications (Table 1). In particular, CGH had previously been applied to TPC-1 cells with the same overall findings [20], even if the lower complexity in that study suggests that our TPC-1 cells acquired several additional chromosome changes in vitro. In accordance, a recent report by van Staveren *et al*. (2007) shows a G-banded karyogram of TPC-1 that is fully compatible with our findings, although the corresponding karyotypic description was not provided [21]. Detailed cytogenetic findings on B-CPAP [22,23] and very recently on the anaplastic cell lines C643, 8505C and HTH74 [24] were also available for comparison. The use of M-FISH in these studies allowed a very refined characterization of several chromosomal markers of unclear origin that we also observed in our samples. We took the high-resolution information reported in these studies into account when generating Table 2. Interestingly, whereas virtually all rearranged chromosomes previously described for cell lines B-CPAP, 8505C and HTH74 are easily identifiable in our data, suggesting these tumor models remain genetically stable in culture, the C643 cell line showed extensive inter-cellular variability and our karyotype shows several dissimilarities to the findings by Lee *et al*. (2007)[24]. This cell line, derived from a highly aggressive metastatic tumor, seems to be genetically unstable and prone to clonal evolution during culture, thus requiring caution when interpreting and comparing results.

Upon describing the genomic background it was also important for us to integrate the findings with known molecular features of the cell lines and to assess their clinical representativeness as tumor models. The meta-analysis of existing cytogenetic and CGH copy number information on non-medulary thyroid tumors showed that papillary carcinomas tend to display simple diploid karyotypes in which rearrangements at 10q11 (*RET *locus) are recurrent events, even if no specific copy number changes could be associated with this histotype (Figure 1). Of the three papillary cell lines, TPC-1 is the only one to harbor a *RET *rearrangement, whereas K1 (GLAG-66) and B-CPAP were recently shown to display the V600E *BRAF *mutation [25]. Interestingly, K1 (GLAG-66) and B-CPAP share several copy number changes (Figure 1), whereas the TPC-1 profile is clearly different from these other two models. Primary follicular carcinomas also tend to display a near-diploid set of chromosomes, but are more complex and display distinctive copy number changes involving mainly gains and losses of whole chromosomes (Figure 2). A recurrent t(2;3) translocation resulting in the *PAX8-PPARγ *chimera can be seen in a subset of samples [26]. The XTC-1 cell line does not harbor this rearrangement, but the CGH profile follows the non-random pattern of most follicular tumors [27], with gains at 1q, 5, 7, 12, 16 and 20. No mutations in *BRAF *or *RAS *have been observed in this cell line [25]. At the more aggressive end of the malignancy spectrum, anaplastic primary carcinomas display correspondingly complex karyotypes with near-triploid chromosomal contents and many aberrations per tumor, even if only few recurrent structural abnormalities are observed (Figure 3). The three anaplastic cell lines follow this pattern (all display complex profiles with noteworthy gains/amplifications at 5p, 8q and 20q), with no detectable rearrangements of *RET *or *PAX8*. Interestingly, cell line 8505C displays a V600E mutation in *BRAF*, whereas *TP53 *mutations can be seen in both C643 and 8505C [25]. It is noteworthy that cell lines 8505C and B-CPAP, which display a near-triploid karyotype with losses at chromosomal region 7q by CGH, show a homozygous pattern for the V600E *BRAF *mutation (*BRAF *is mapped to 7q34). This indicates that the two cytogenetically normal chromosomes 7 observed in both cell lines carry the mutation. Rather than resulting from two independent mutational events, this finding suggests that the *BRAF *mutation occurred in one chromosome 7 that later was duplicated and that the 7q with the wild type *BRAF *was deleted.

Unfortunately, other candidate genes are difficult to highlight in this panel of cell lines, as regions recurrently affected by copy number gains and/or losses always involved large DNA segments. It should also be noted that continued cell culture tends to generate clonal evolution in vitro, and in particular the tetraploidization phenomena seems to occur frequently in cell lines over time (FB2 and K1 being good examples). Even if these events may generate considerable karyotypic differences in cell line stocks in different laboratories, one would expect that the biologically relevant oncogenic rearrangements characterizing each cell line are maintained in the different subpopulations. Classical cytogenetic analysis will efficiently detect such clonal divergence, although it requires considerable time and expertise. An alternative and less time-consuming method to ensure cell line identity is genotype analysis using Short Tandem Repeat (STR) markers [24,25], although clonal evolution will not be detected using this approach. Indeed, a recently published survey of 40 presumptive thyroid cell lines using STR markers showed that only 23 cell lines displayed unique profiles consistent with thyroid origin (encompassing the seven unique cell lines in the current study), with the remaining cell lines being derivatives or cross-contaminants from different origins, such as melanoma and colon cancer cell lines [28].

## Conclusion

We report the genetic background of seven independent thyroid cell lines of papillary, follicular or anaplastic origin, highlighting distinctive chromosomal markers as well as common genomic regions of interest for future studies. Importantly, the literature review indicates these cellular models display genetic alterations representative of those found in clinical tumors of the correspondent histotype. Our findings further remind us that cell line contamination happens more frequently than one would like to admit, and that karyotyping and/or genotyping are efficient methods for clonal analysis and determination of cell line identity. We therefore recommend that karyotype and genotype analysis be performed from early passages and on a regular basis in every laboratory working with cancer cell lines to help interpret the genetic data, to detect in vitro clonal evolution with time, and to ensure cell line authenticity.

## Competing interests

The authors declare that they have no competing interests.

## Authors' contributions

FRR carried out the karyotyping and CGH experiments, performed literature data mining and analysis, and drafted the manuscript. AMM and ASR performed cell line culture and DNA extraction. MRT designed and coordinated the study, assisted with analysis, and contributed to manuscript writing. All authors read and approved the final manuscript.

## Pre-publication history

The pre-publication history for this paper can be accessed here:



## Supplementary Material

Additional file 1 **Genetic findings in cell line TPC-1**. Representative karyogram (a) and copy number profile (b) of papillary thyroid carcinoma cell line TPC-1. For a complete description of the genomic findings refer to Table 2. Click here for file

Additional file 2 **Genetic findings in cell line FB2**. Representative karyogram (a) and copy number profile (b) of papillary thyroid carcinoma cell line FB2 (derived from cell line TPC-1). For a complete description of the genomic findings refer to Supplementary Table 1. Click here for file

Additional file 3 **Genetic findings in cell line K1**. Representative karyogram (a) and copy number profile (b) of papillary thyroid carcinoma cell line K1 (derived from cell line GLAG-66). For a complete description of the genomic findings refer to Table 2. Click here for file

Additional file 4 **Genetic findings in cell line B-CPAP**. Representative karyogram (a) and copy number profile (b) of papillary thyroid carcinoma cell line B-CPAP. For a complete description of the genomic findings refer to Table 2. Click here for file

Additional file 5 **Genetic findings in cell line XTC-1**. Representative karyogram (a) and copy number profile (b) of follicular thyroid carcinoma cell line XTC-1. For a complete description of the genomic findings refer to Table 2. Click here for file

Additional file 6 **Genetic findings in cell line HTH74**. Representative karyogram (a) and copy number profile (b) of anaplastic thyroid carcinoma cell line HTH74. For a complete description of the genomic findings refer to Table 2. Click here for file

Additional file 7 **Genetic findings in cell line C643**. Representative karyogram (a) and copy number profile (b) of anaplastic thyroid carcinoma cell line C643. For a complete description of the genomic findings refer to Table 2. Click here for file

Additional file 8 **Genetic findings in cell line 8505C**. Representative karyogram (a) and copy number profile (b) of anaplastic thyroid carcinoma cell line 8505C. For a complete description of the genomic findings refer to Table 2. Click here for file

Additional file 9 **Comparison of findings in TPC-1 and FB2**. Partial karyograms of the shared chromosomal aberrations displayed by TPC-1 and FB2 cells. The derivatives of the complex t(1;10;21) are depicted first, followed by the del(7)(q22q31) and the i(8)(q10). The arrows indicate the final location of the RET/PTC1 rearrangement to the derivative 21. Click here for file

Additional file 10 **Genetic findings in TPC-1 and FB2 cell lines**. Karyotype and CGH descriptions of cell lines TPC-1 and FB2, highlighting shared aberrations indicative of their common origin. Click here for file
