# Supplementary figures and images for: Conventional and molecular cytogenetics of human non-medullary thyroid carcinoma: characterization of eight cell line models and review of the literature on clinical samples
Source: BMC Cancer. 2008 Dec 16;8:371. doi: 10.1186/1471-2407-8-371 (PMC2651892; doi:10.1186/1471-2407-8-371)

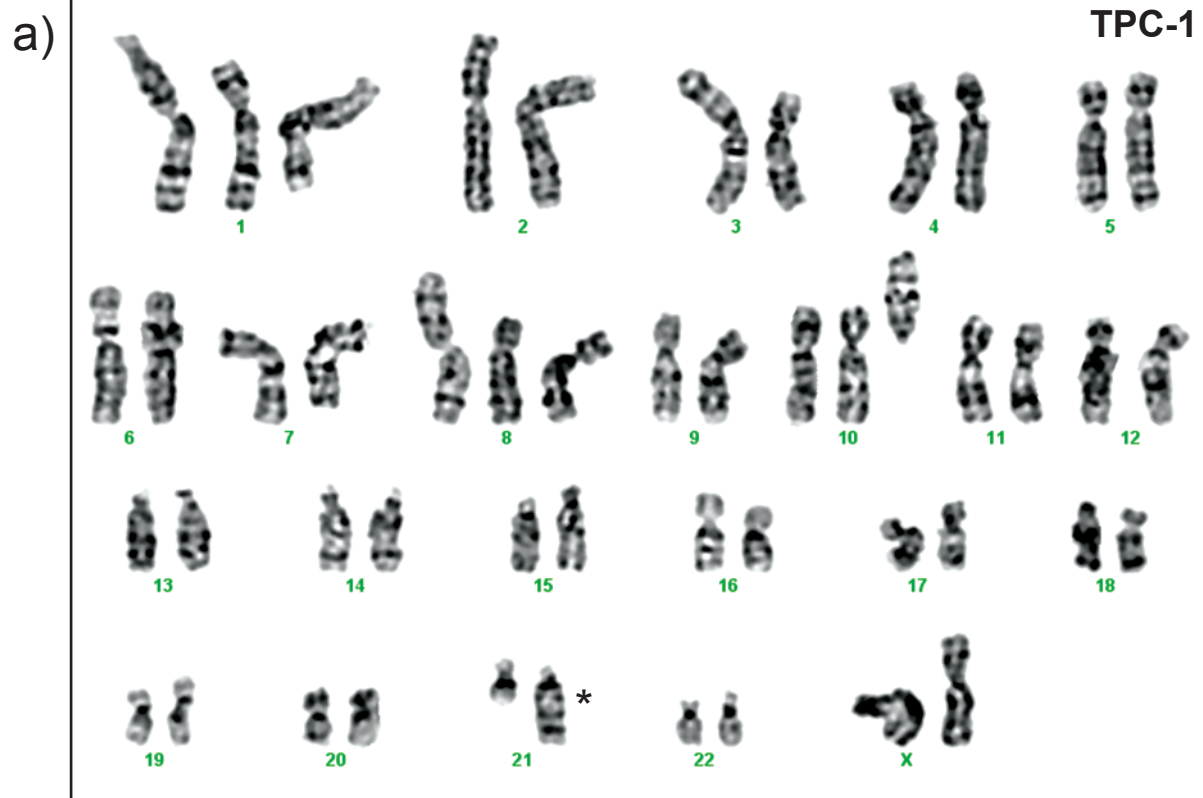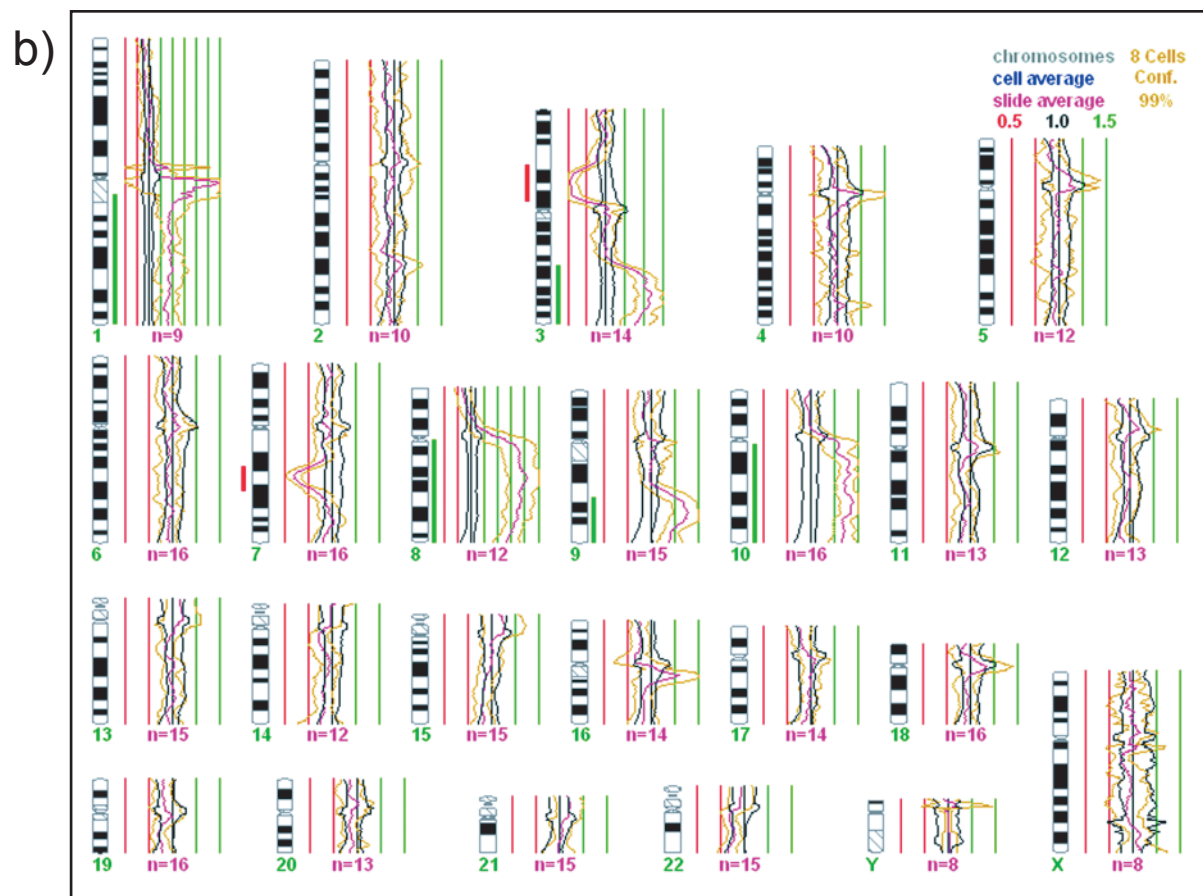

Supplement: Additional file 1 — Genetic findings in cell line TPC-1. Representative karyogram (a) and copy number profile (b) of papillary thyroid carcinoma cell line TPC-1. For a complete description of the genomic findings refer to Table 2. [file 1471-2407-8-371-S1.pdf]

a)

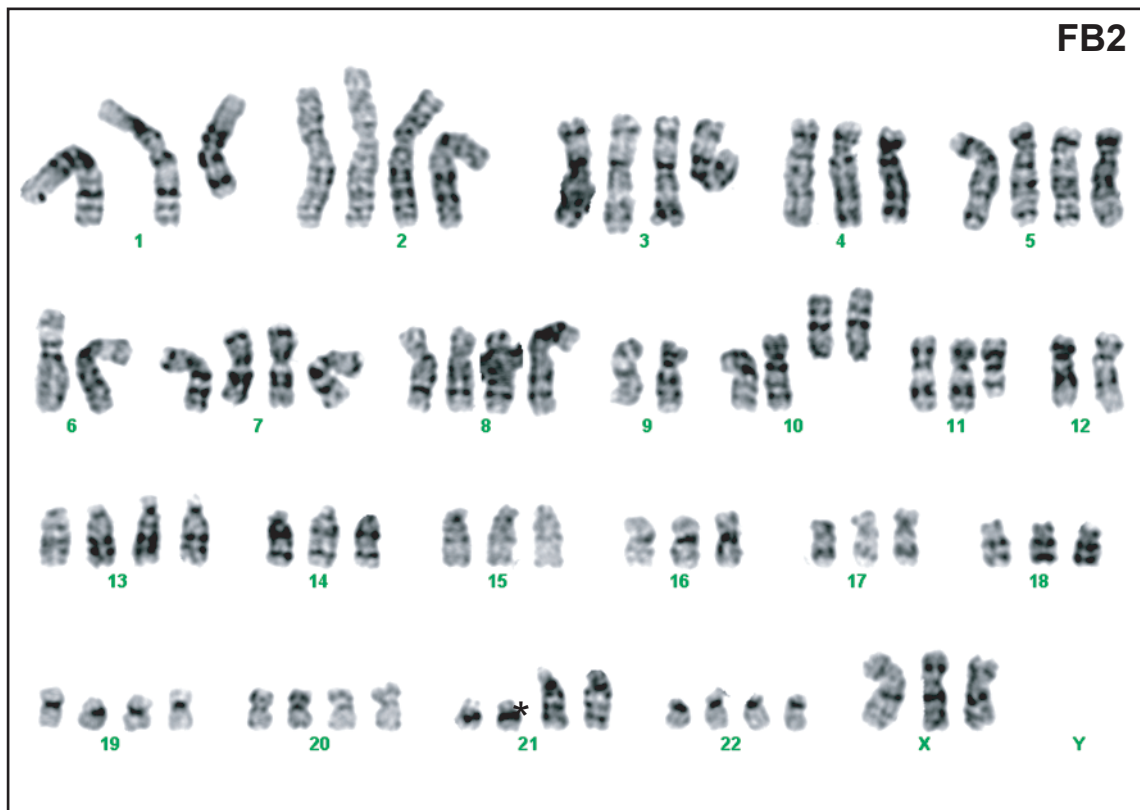

b)

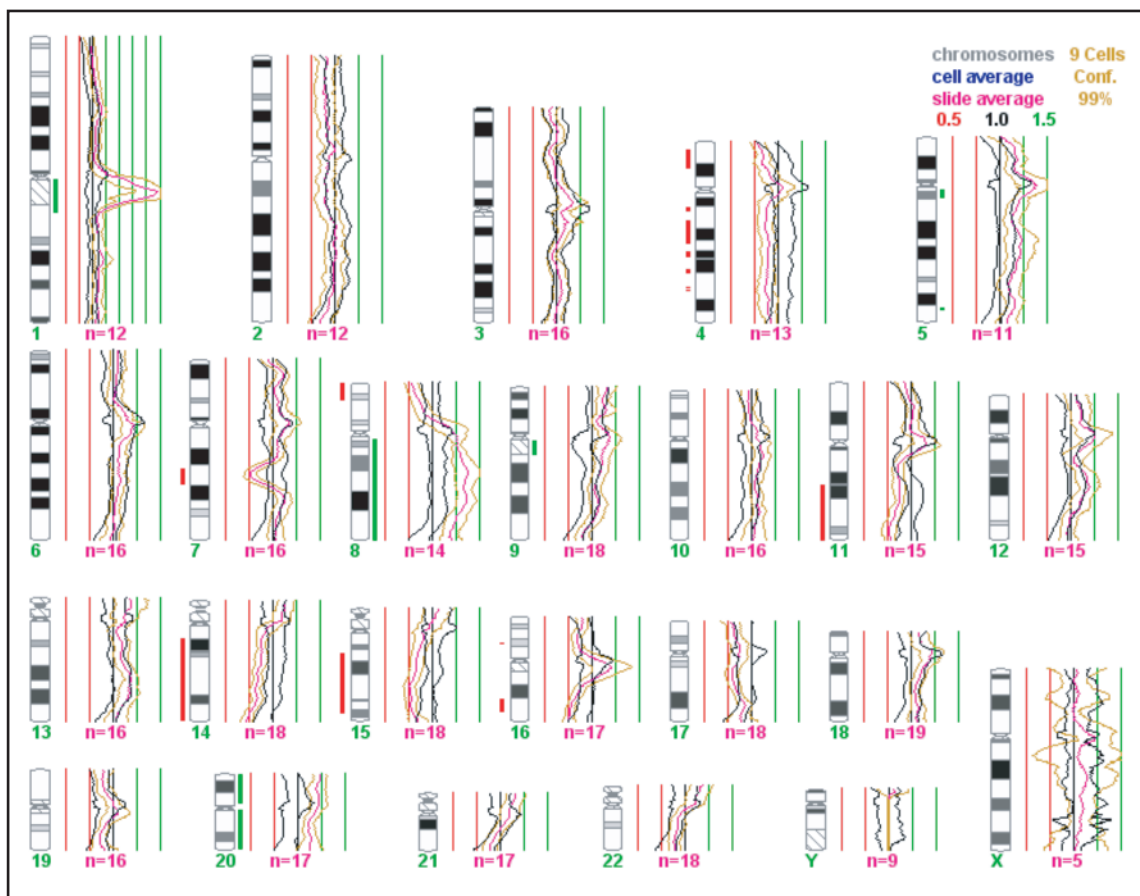

Supplement: Additional file 2 — Genetic findings in cell line FB2. Representative karyogram (a) and copy number profile (b) of papillary thyroid carcinoma cell line FB2 (derived from cell line TPC-1). For a complete description of the genomic findings refer to Supplementary Table 1. [file 1471-2407-8-371-S2.pdf]

a)

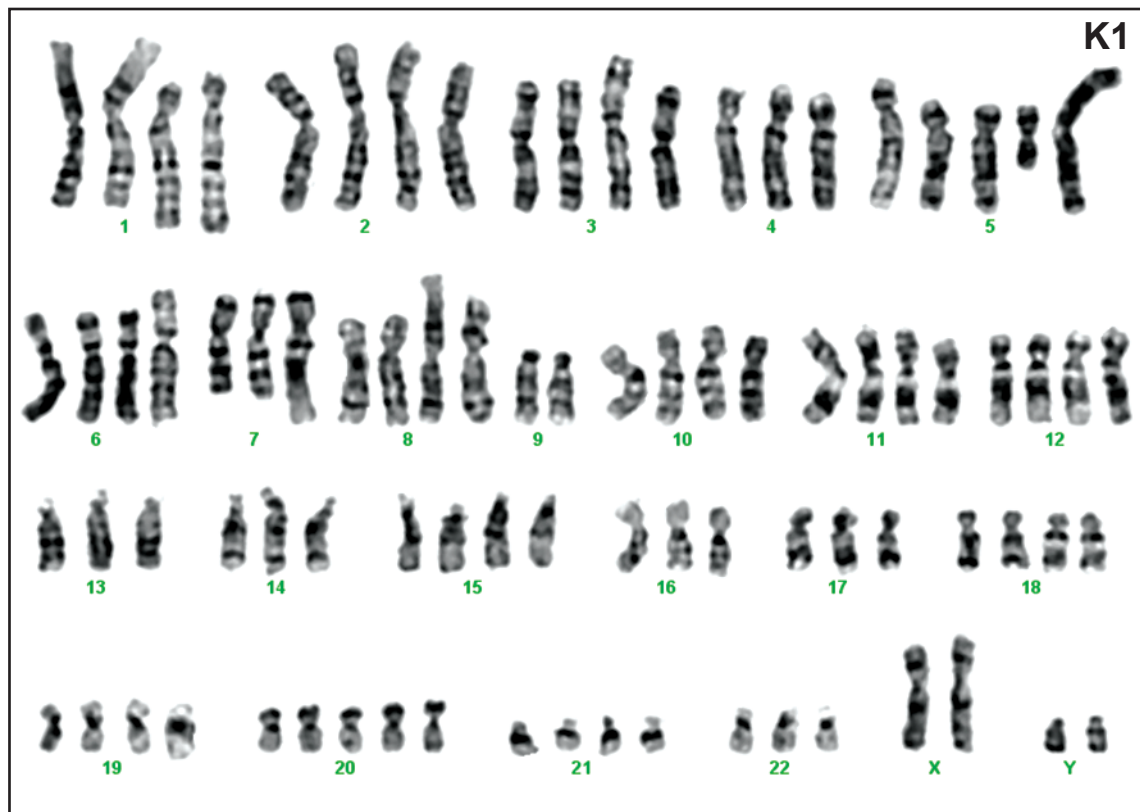

b)

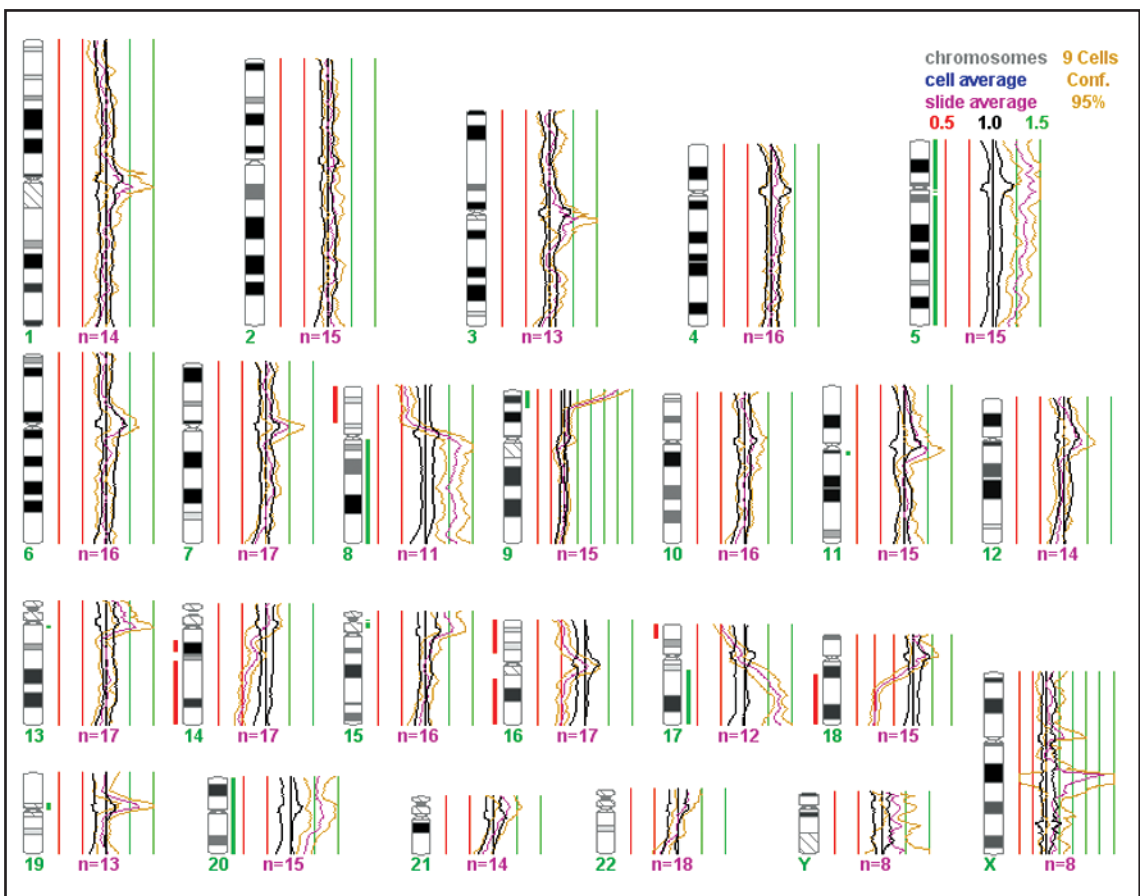

Supplement: Additional file 3 — Genetic findings in cell line K1. Representative karyogram (a) and copy number profile (b) of papillary thyroid carcinoma cell line K1 (derived from cell line GLAG-66). For a complete description of the genomic findings refer to Table 2. [file 1471-2407-8-371-S3.pdf]

a)

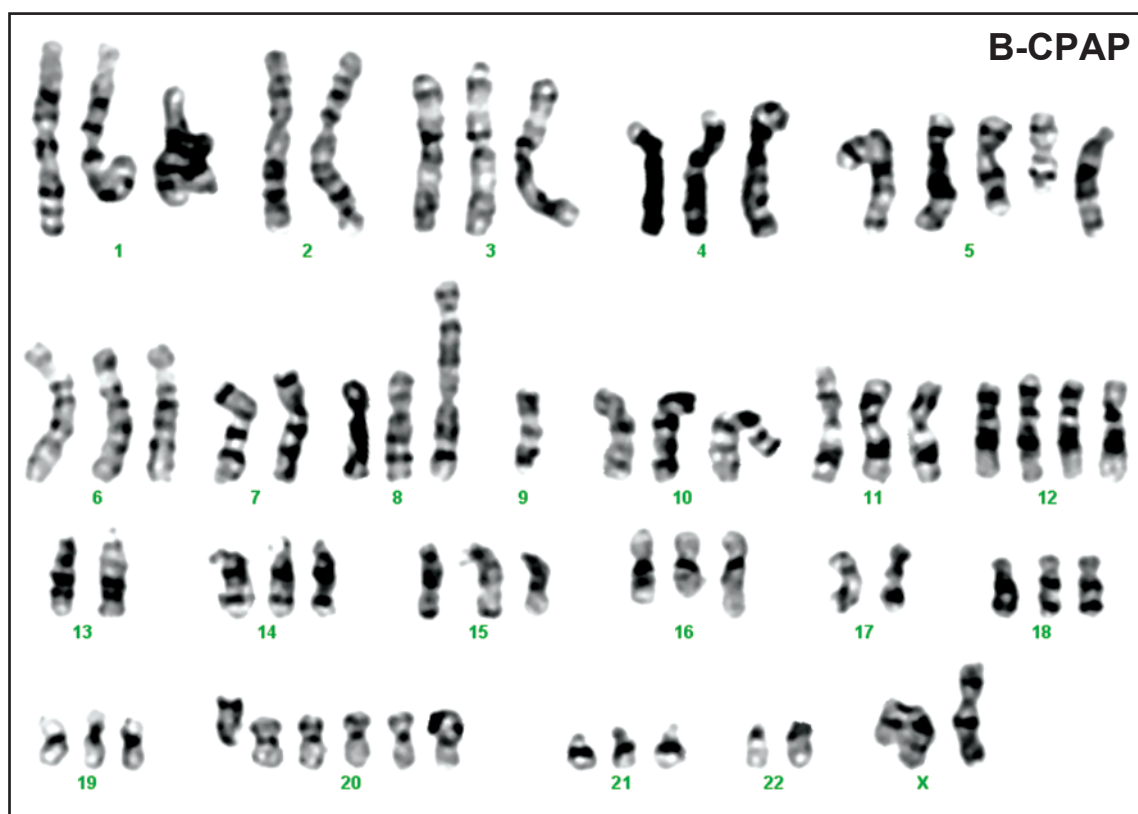

b)

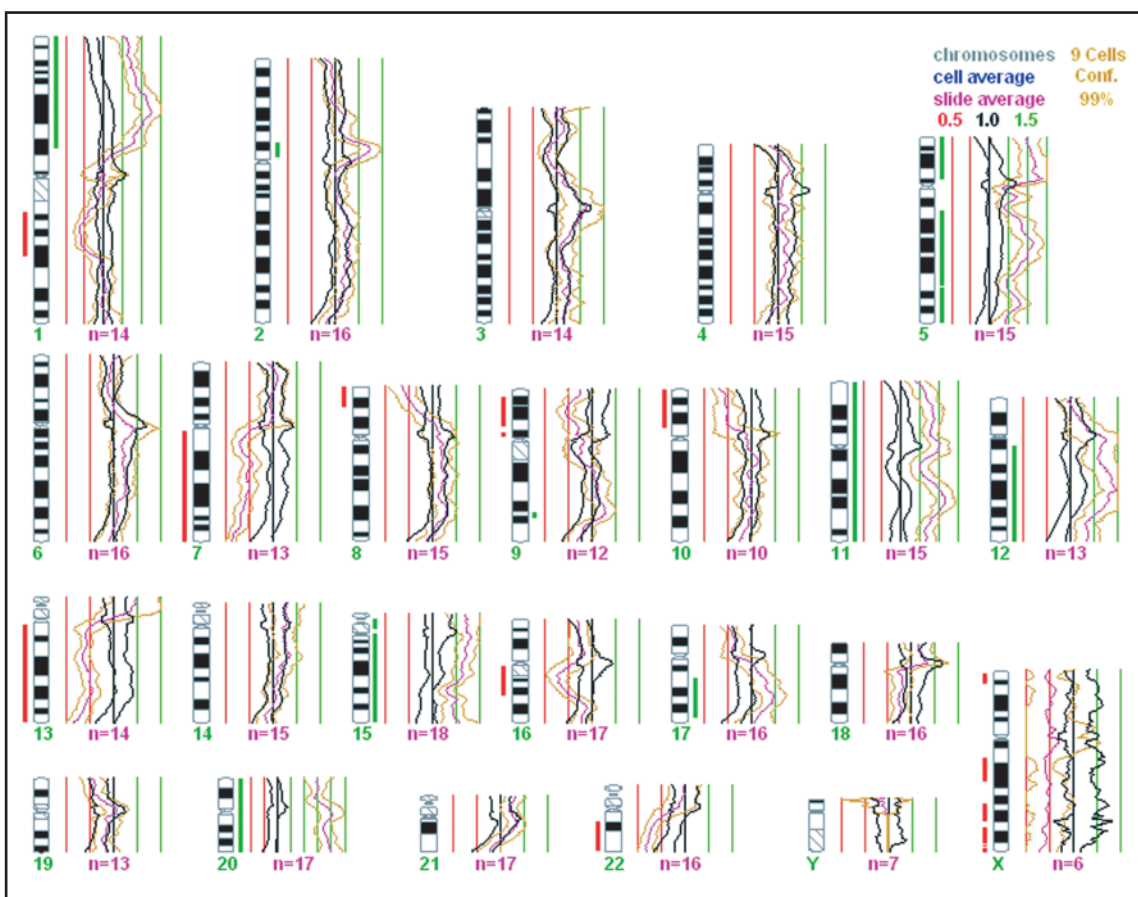

Supplement: Additional file 4 — Genetic findings in cell line B-CPAP. Representative karyogram (a) and copy number profile (b) of papillary thyroid carcinoma cell line B-CPAP. For a complete description of the genomic findings refer to Table 2. [file 1471-2407-8-371-S4.pdf]

a)

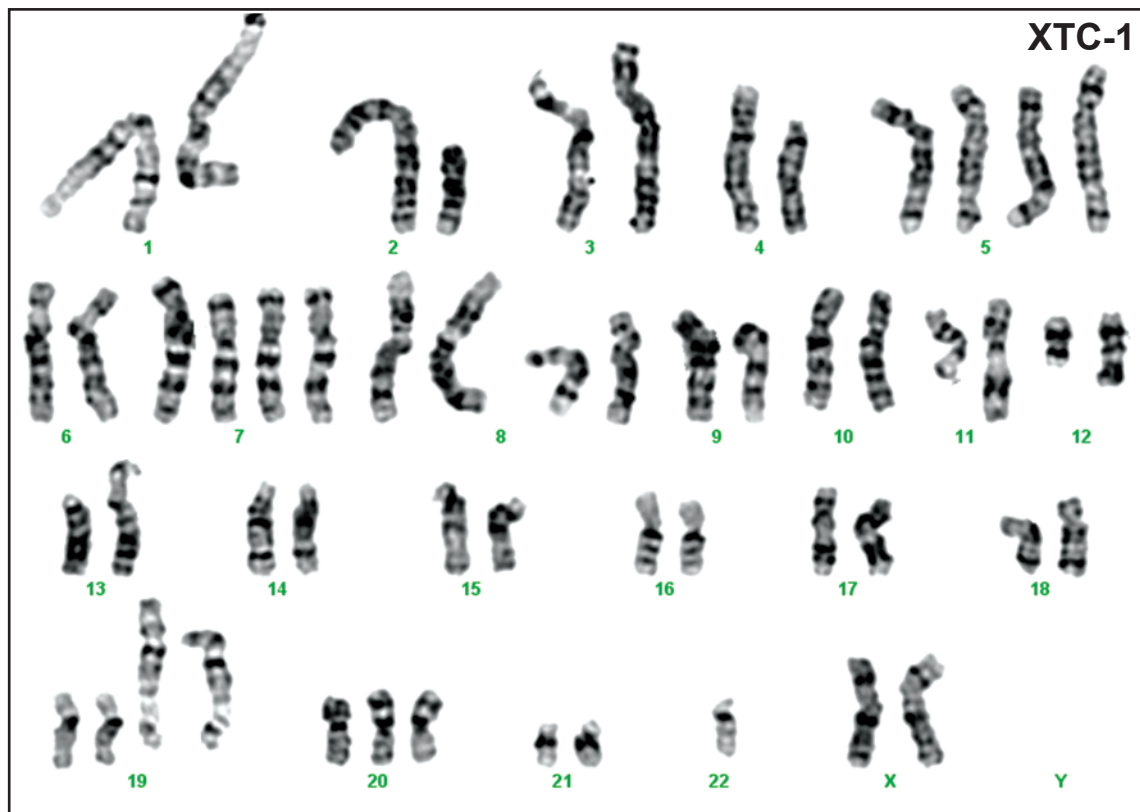

b)

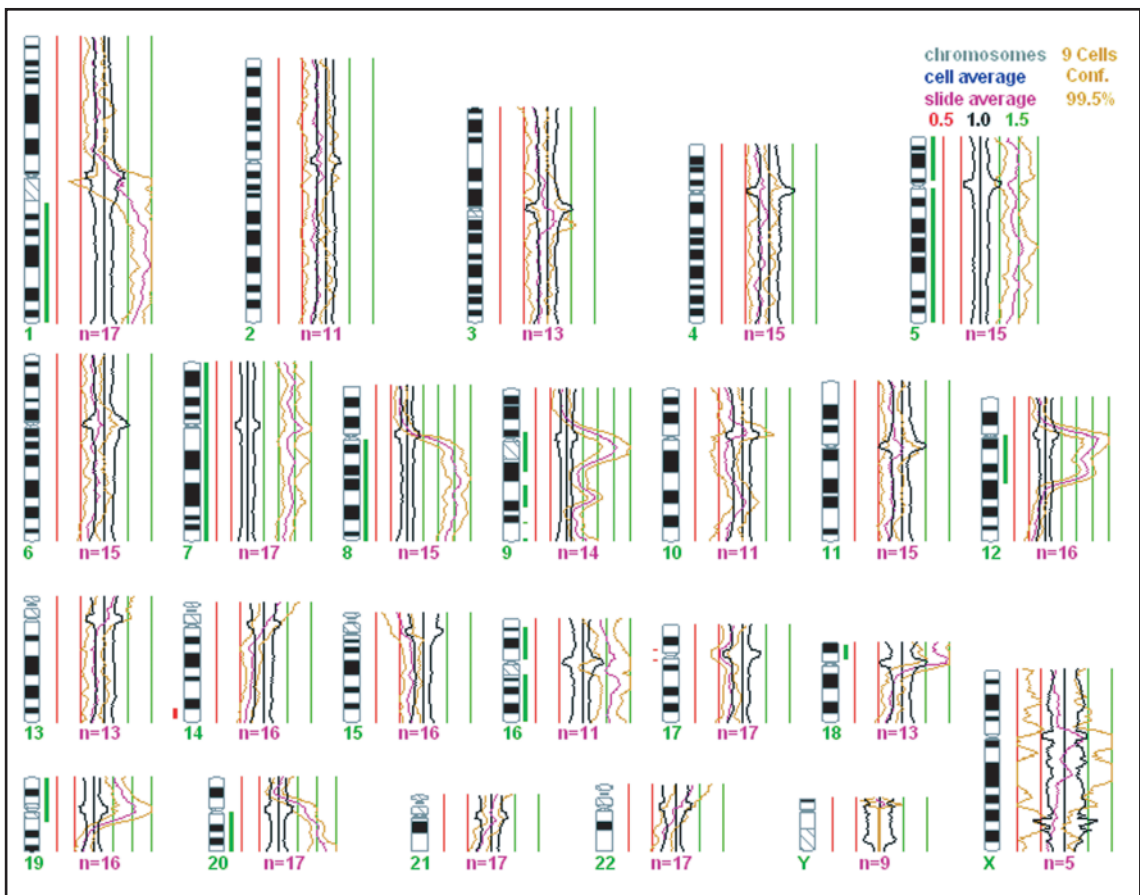

Supplement: Additional file 5 — Genetic findings in cell line XTC-1. Representative karyogram (a) and copy number profile (b) of follicular thyroid carcinoma cell line XTC-1. For a complete description of the genomic findings refer to Table 2. [file 1471-2407-8-371-S5.pdf]

a)

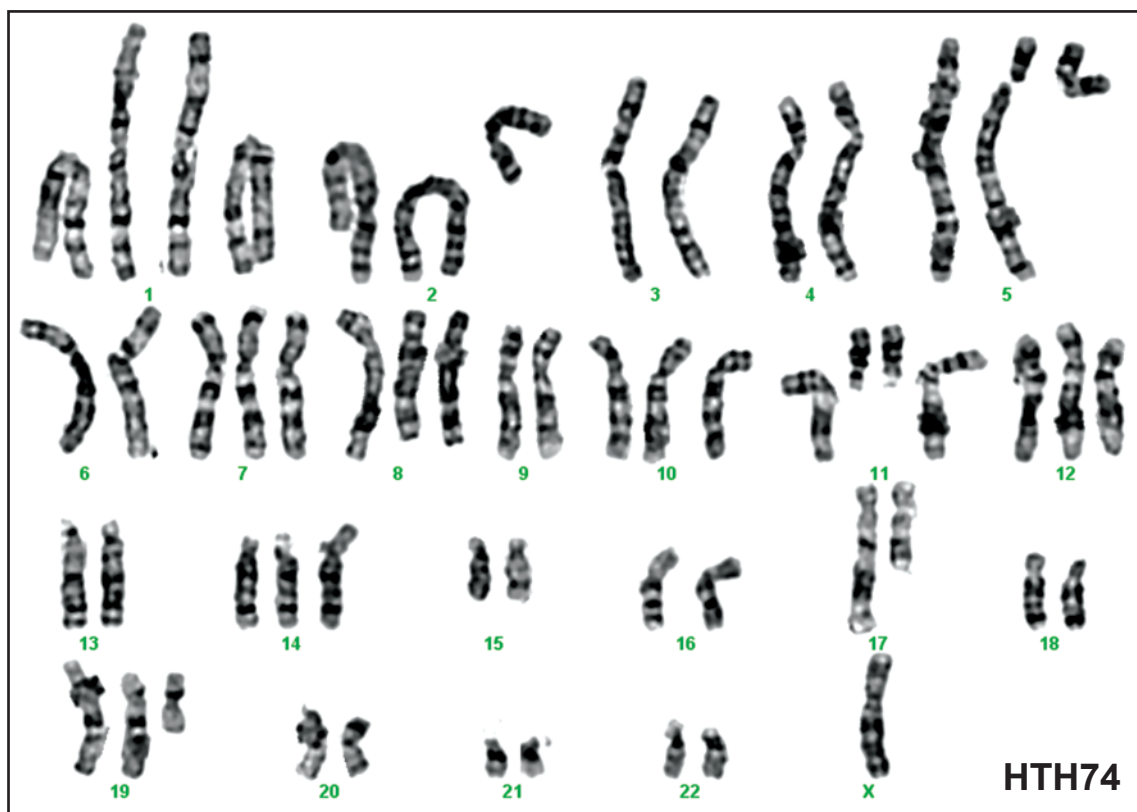

b)

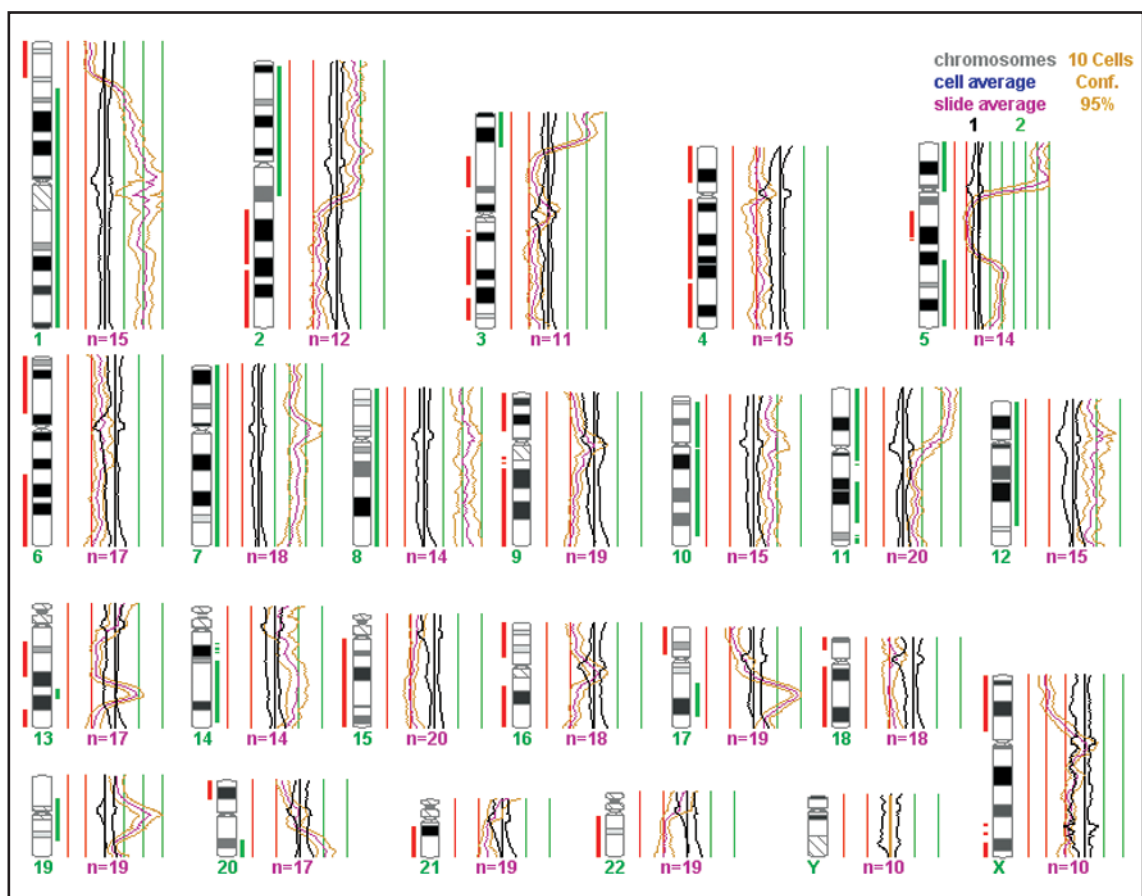

Supplement: Additional file 6 — Genetic findings in cell line HTH74. Representative karyogram (a) and copy number profile (b) of anaplastic thyroid carcinoma cell line HTH74. For a complete description of the genomic findings refer to Table 2. [file 1471-2407-8-371-S6.pdf]

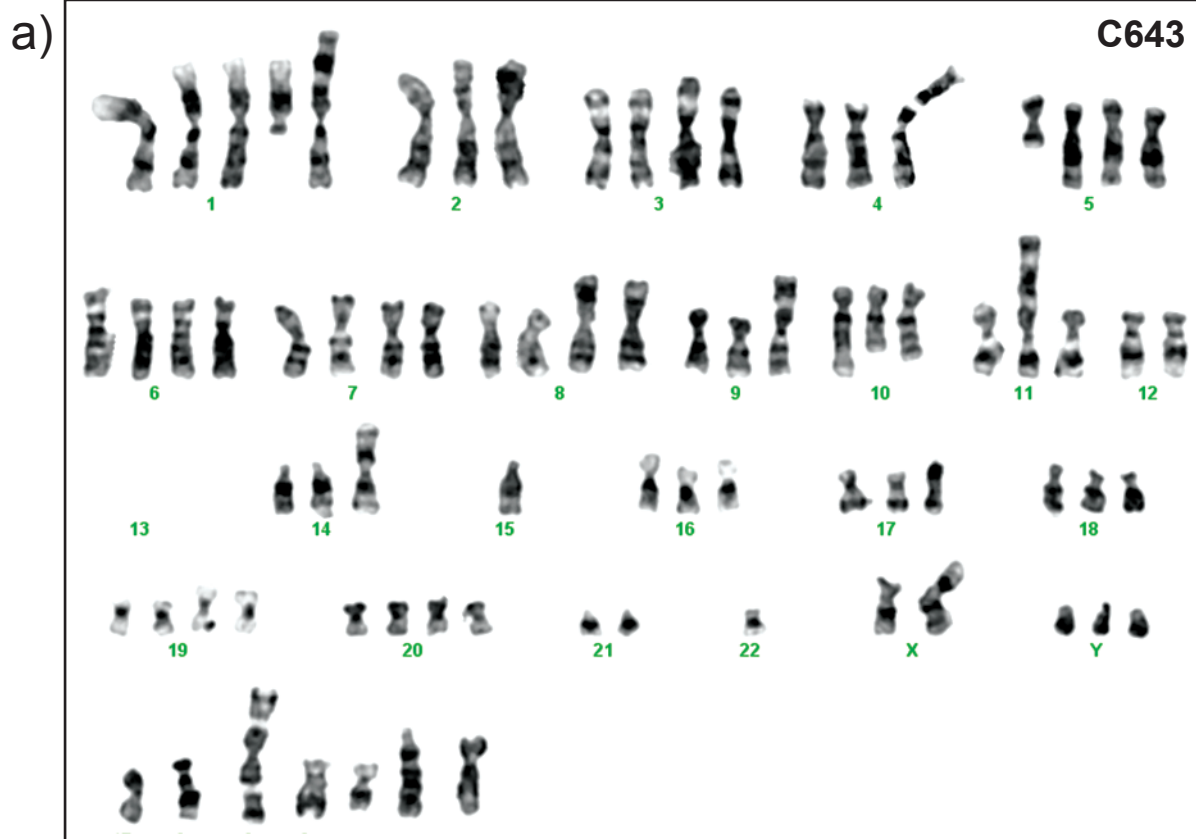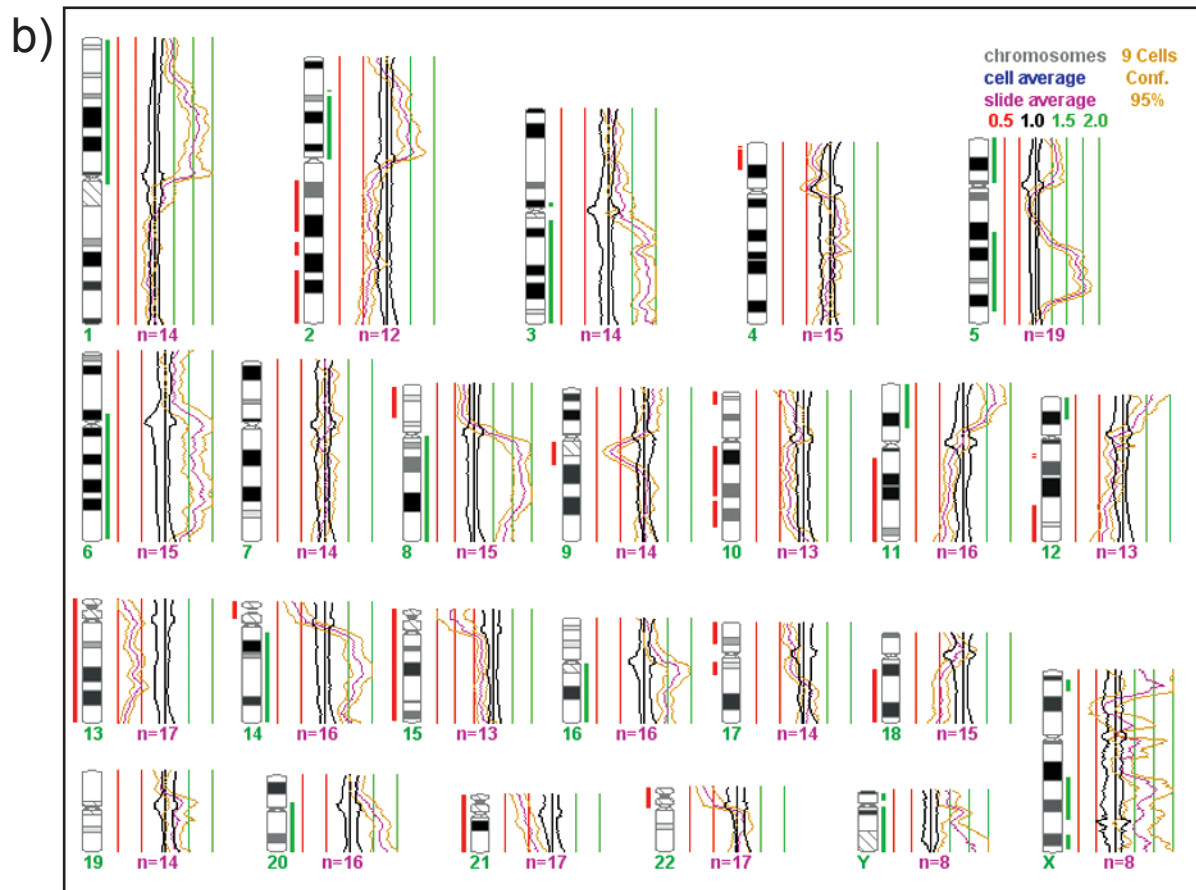

Supplement: Additional file 7 — Genetic findings in cell line C643. Representative karyogram (a) and copy number profile (b) of anaplastic thyroid carcinoma cell line C643. For a complete description of the genomic findings refer to Table 2. [file 1471-2407-8-371-S7.pdf]

a)

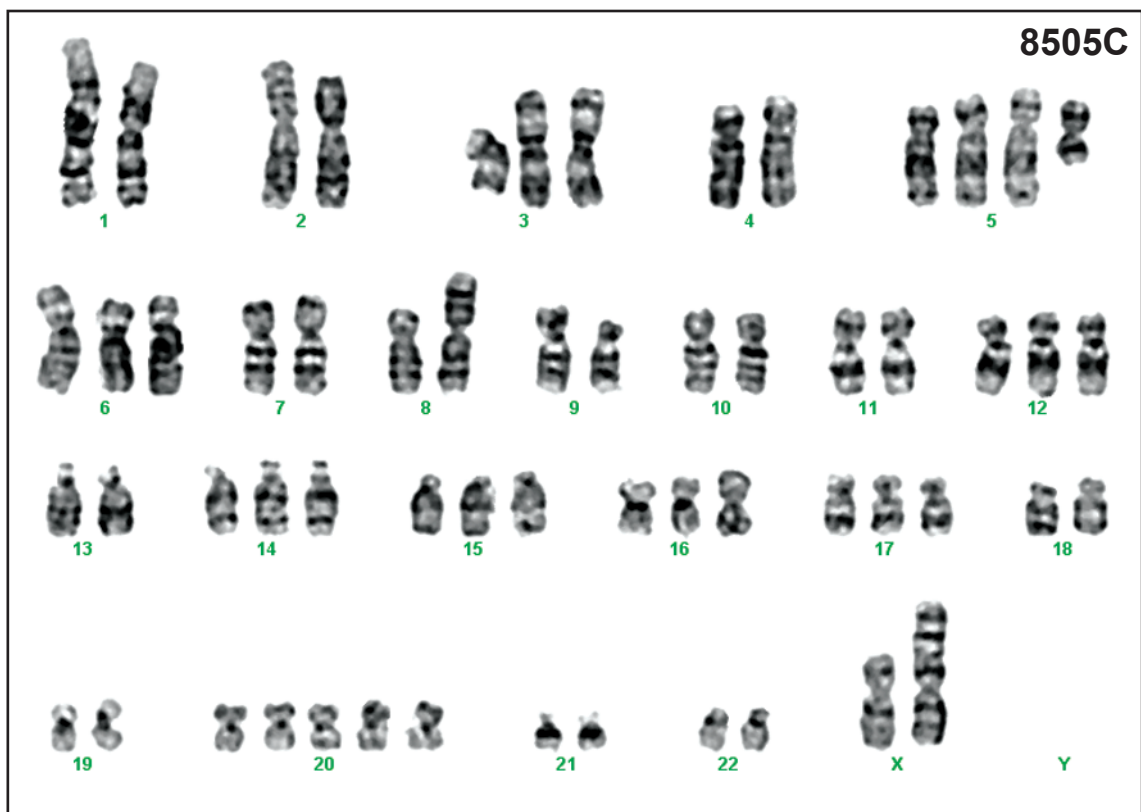

b)

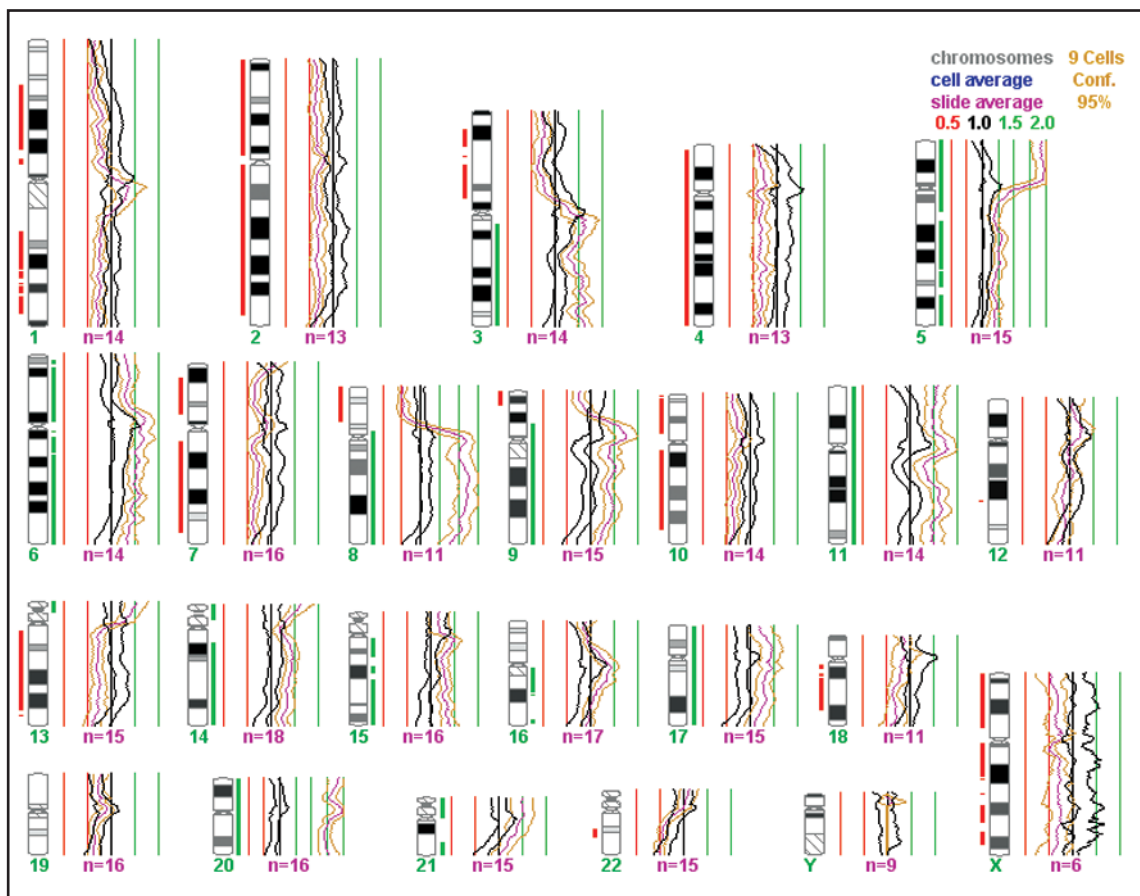

Supplement: Additional file 8 — Genetic findings in cell line 8505C. Representative karyogram (a) and copy number profile (b) of anaplastic thyroid carcinoma cell line 8505C. For a complete description of the genomic findings refer to Table 2. [file 1471-2407-8-371-S8.pdf]

TPC-1

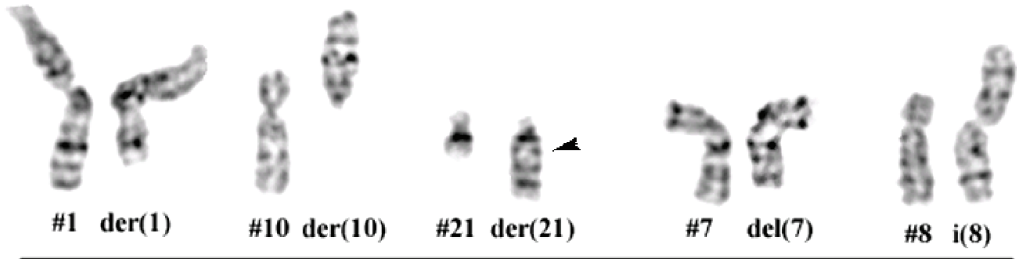

FB2

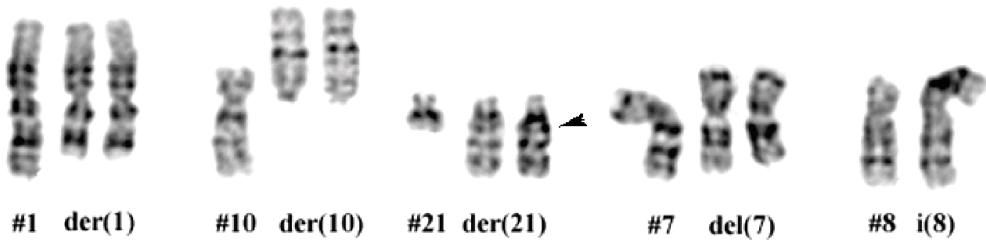

Supplement: Additional file 9 — Comparison of findings in TPC-1 and FB2. Partial karyograms of the shared chromosomal aberrations displayed by TPC-1 and FB2 cells. The derivatives of the complex t(1;10;21) are depicted first, followed by the del(7)(q22q31) and the i(8)(q10). The arrows indicate the final location of the RET/PTC1 rearrangement to the derivative 21. [file 1471-2407-8-371-S9.pdf]
