# Supplementary material for: Conventional and molecular cytogenetics of human non-medullary thyroid carcinoma: characterization of eight cell line models and review of the literature on clinical samples
Source: BMC Cancer. 2008 Dec 16;8:371. doi: 10.1186/1471-2407-8-371 (PMC2651892; doi:10.1186/1471-2407-8-371)
Supplement: Additional file 10 — Genetic findings in TPC-1 and FB2 cell lines. Karyotype and CGH descriptions of cell lines TPC-1 and FB2, highlighting shared aberrations indicative of their common origin. [file 1471-2407-8-371-S10.pdf]

Supp Table 1 - Genetic findings in TPC-1 and FB2 cell lines

Cell line	Karyotype	CGH
TPC-1	49,XX,**der(1)t(1;10;21)(1pter→1q31::21q21→21qter)**, +der(1)t(1;3)(p13;p21),der(3)t(3;9)(p11;q22),**del(7)(q22q31)**, +**i(8)(q10)**,**der(10)t(1;10;21)(10q11.2→10pter::1q31→1qter)**, +der(10)inv(10)(q11.2q21)t(3;10)(q25;p11),**der(21)t(1;10;21) (21pter→21q21::10q21.2→10q11.2::10q21.2→ 10qter)**[25]	rev ish enh(1)(q),dim(3)(p12p21),enh(3)(q24q29), **dim(7)(q21q31)**,**amp(8)(q)**,enh(9)(q22q34),enh(10)(q)
FB2	82~87,XXXX,**der(1)t(1;10;21)(1pter→1q31::21q21→21qter)**x2, **-4**,**del(7)(q22q31)**x2,**i(8)(q10)**,**der(10)t(1;10;21) (10pter->10p15::10q11.2→ 10p15::1q31→ 1qter)**x2,-11,-14,-15,-16,-17, +20,**der(21)t(1;10;21)(21pter→ 21q21::10q21.2→ 10q11.2::10q21.2→10qter)**x2[20]/82~87,idem, der(6)t(1;6)(p32;q15)[5]/82~87, idem,der(11)t(11;17)(q14;q24), add(12)(q24)[6]	rev ish dim(4),**dim(7)(q22q31)**,dim(8)(p21p23),**enh(8)(q)**, dim(11)(q14q25),dim(14)(q),dim(15)(q),dim(16)(p11p13), dim(16)(q),enh(20)

Karyotype and CGH descriptions follow the ISCN 2005 guidelines. Shared chromosomal aberrations are depicted in bold.
